# Denaturing SUMO Immunoprecipitation From Mitotic Cells

**DOI:** 10.21769/BioProtoc.5648

**Published:** 2026-04-05

**Authors:** Alexandra K. Walker, Alexander J. Lanz, Joanna R. Morris

**Affiliations:** Department of Cancer and Genomic Sciences, School of Medical Sciences, College of Medicine and Health, and Birmingham Centre for Genome Biology, University of Birmingham, Birmingham, UK

**Keywords:** SUMO1, SUMO2/3, Mitosis, Nocodazole, Denature, Immunoprecipitation, Western blot

## Abstract

Small ubiquitin-related modifiers (SUMOs) are covalently conjugated onto the proteome and serve as signaling molecules in many aspects of eukaryotic cell biology, from *S. cerevisiae* and *C. elegans* to *H. sapiens*. The conjugatable SUMO variants, SUMO1 and the almost identical SUMO2 and SUMO3 (designated SUMO2/3), are processed by an E1(SAE1:SAE2)-E2(UBC9)-E3 enzyme cascade to produce SUMO-modified proteins. The prerogative of the SUMO biology field is to identify and study the specific proteins undergoing SUMOylation, which grants us insights into the biological pathway of interest. This protocol was developed using the human osteosarcoma cell line U2OS to enable the investigation of SUMO conjugates in mitosis, the cell division phase of the cell cycle. We enrich the cell population for mitotic cells, which are isolated and subjected to stringent lysis conditions involving a high concentration of SDS and DTT in RIPA buffer, to promote complete protein denaturation. The lysates in high SDS RIPA buffer are diluted to reduce the overall SDS concentration and undergo conventional immunoprecipitation using SUMO1- or SUMO2/3-specific antibodies bound to protein A/G agarose beads. The samples are then compatible with downstream readouts such as western blots and mass spectrometry. This protocol detects endogenous SUMOylated proteins and avoids exogenous SUMO overexpression, which can alter SUMO conjugate formation. Furthermore, this denaturing protocol ensures only SUMOylated proteins are immunoprecipitated, and not their interactors.

Key features

• Purifies endogenous SUMO-modified proteins by building on Becker et al. [1].

• Enriches and isolates cells in mitosis using nocodazole and mitotic shake-off.

• 1% SDS RIPA lysis promotes robust denaturation ahead of SUMO-specific immunoprecipitation.

• Compatible with downstream readouts such as western blots and mass spectrometry.

## Graphical overview


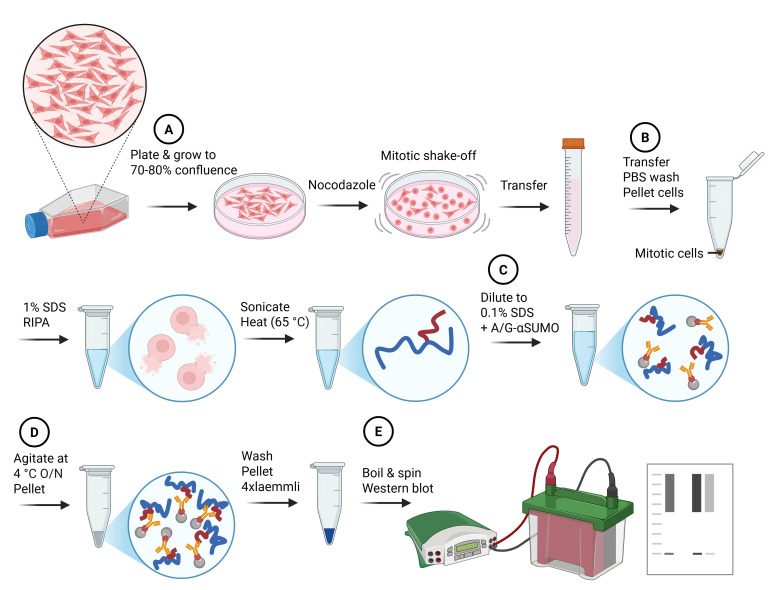


**Denaturing small ubiquitin-related modifiers (SUMO) immunoprecipitation workflow.** Major steps of the protocol and the impacts on cells and proteins are summarized in cartoon style. Letters in circles correspond to the protocol subsections (below). Blue lines indicate denatured proteins, and red lines indicate denatured SUMO protein conjugates.

## Background

The small ubiquitin-related modifier (SUMO) can be covalently transferred onto proteins, altering their subcellular localization, protein–protein interactions, and/or activity. The conjugation of SUMO moieties onto substrates occurs via the processive actions of a unique E1 activation enzyme (composed of a heterodimer containing SAE1/AOS1 and SAE2/UBA2) and an E2 conjugating enzyme (UBC9), with many E3 ligases assisting [2]. Within higher eukaryotes, there are three variants of conjugatable SUMO: SUMO1, SUMO2, and SUMO3. SUMO2 and SUMO3 share a 97% sequence identity and are virtually indistinguishable [3]; as such, they are often designated SUMO2/3. SUMO1 and SUMO2/3 share many substrates, but proteomic studies have revealed that SUMO2/3-modified substrates do not exclusively overlap with those modified by SUMO1 [1,4]. The divergence between SUMO1 and SUMO2/3 is particularly evident in mitosis, where SUMO1 localizes to the mitotic spindle, whereas SUMO2/3 co-occupies areas associated with centromeres and condensed chromosomes [5].

SUMOylation has emerged as a vital regulator of mitosis, with defects in mitotic progression and chromosome segregation observed upon deficiency, depletion, or chemical inhibition of core components within the SUMO conjugation machinery [6–10]. While SUMO conjugation is required for efficient and accurate mitotic progression, it is notable that global SUMO conjugates are lower during this phase of the cell cycle [5], which can make identifying alterations in SUMOylation during mitosis challenging. Therefore, obtaining a large and relatively “pure” mitotic population is essential to accurately pinpoint mitotic SUMOylation events that are not skewed by asynchronous contaminants. Due to the short duration of this phase of the cell cycle (approximately 1 h in RPE, HeLa and MCF10A) [11], cell synchronization is commonly used to obtain sufficient cell numbers to study. This has classically been achieved using microtubule poisons such as nocodazole [12,13] and mitotic shake-off [14].

The identification of SUMO conjugates requires cell lysates to be prepared under denaturing conditions. This encourages the stability of the SUMO modification by inhibiting the actions of SUMO-specific proteases/isopeptidases, as well as reducing the co-precipitation of non-covalent SUMO interactors [15]. The simplest method of identifying SUMOylated proteins is through the detection of a mobility shift on denaturing gels that is sensitive to SUMO-specific proteases and/or disruption to SUMO conjugation machinery. Whilst this may be a valid starting point for identifying a SUMO substrate, it is indirect, biased toward abundant or highly modified targets, and only speaks to the modification of one protein. To increase the abundance of SUMO-modified substrates and to enable the identification of SUMOylated sites on target proteins, affinity purification with overexpressed SUMO constructs has frequently been employed. The use of 6xHis-tagged exogenous proteins and Ni-NTA resin is particularly used due to its compatibility with denaturing conditions [4,15–20]. However, the use of overexpression is an inherent issue, as it intrinsically alters the balance of SUMO variants (i.e., SUMO1 and SUMO2/3), which has the potential to alter their usage [7,21]. A further caveat to using overexpression is the prerequisite for cells to have an acceptable degree of transfectability. This can limit the cell lines and types of samples that can be used to identify SUMOylated substrates.

A number of methods have been developed to enrich SUMO conjugates under endogenous conditions [1,22–28]. Many of these techniques rely on mass spectrometry–based proteomics to identify SUMO moieties or remnants on peptides [22–26]. These methods are integral to our understanding of SUMO biology, enabling the site-specific identification of SUMOylation on substrates. However, the main limitation in adopting these approaches for more frequent use, among the wider molecular biology community, is the high degree of specialism they require. The level of information that these techniques are able to acquire may also lie beyond the scope of the specific research question under investigation.

The enrichment of endogenous SUMOylated proteins (as opposed to peptides) has been described using SUMO-traps [alternatively named SUMO binding entities (SUBEs)] [28] and antibodies [1]. Both approaches require less specialism to perform, and protein identification can occur through targeted western blotting or mass spectrometry. SUMO-traps can capture endogenous poly-SUMOylated proteins using a GST-fusion protein bearing tandem SUMO interacting motif repeats from the SUMO E3 RNF4. However, these are not suited to the identification of mono-SUMOylated proteins and consequently bias results toward SUMO2/3-containing substrates. Additionally, as it is currently presented in the literature, this assay is performed under non-denaturing conditions, and therefore, non-covalent SUMO interactors and interacting proteins can also be co-precipitated with SUMOylated substrates [28]. Becker et al. [1] described a procedure that used commercially available antibodies and denaturing conditions to enrich SUMOylated substrates. The main benefits of this technique derive from its ease of use, accessibility of reagents, and relative affordability. Due to the initial denaturation of lysates, misidentification of non-covalent interactors should be avoided. However, the reliance of this assay on antibodies is a major limitation. Antibodies are inherently limited by the specificity of their antigen and may not capture all SUMO substrates or bind other SUMO paralogs to those intended. Batch-to-batch variation within polyclonal antibodies can also contribute to variation in results. Lastly, if a significant enough enrichment is not achieved through immunoprecipitation, this assay may struggle to detect modifications on lowly expressed or modified proteins.

Here, we describe an optimized procedure to assess SUMO conjugation in mitosis. This assay was successfully employed to characterize an alteration in SUMO variant usage during mitosis when an acetyl mimic of the SUMO E1 component SAE2 (SAE2-K164Q) was expressed in cells [7]. Building on the assay described by Becker et al. [1], cells are enriched for mitosis using nocodazole and further isolated via a mitotic shake-off. Lysis and denaturation then proceed, using a high-percentage SDS buffer and treatment with DTT. The denatured lysate is diluted tenfold with a 0% SDS lysis buffer before a standard immunoprecipitation with commercial antibodies. This method bears the same underlying disadvantages as the Becker et al. technique, described above, with some additional caveats: the requirement for a large number of cells and the use of agents to synchronize cells before harvesting. The benefits of using this assay over other techniques include its ease of use, high adaptability to investigate specific research questions, lack of requirement for specialist equipment, and the absence of SUMO variant imbalance generated by overexpression.

## Materials and reagents

**Biological materials**

1. U-2 OS, *Homo sapiens* bone osteosarcoma (ATCC, HTB-96)

**Reagents**

1. Dulbecco’s modified Eagle’s medium, high glucose (Sigma-Aldrich, catalog number: D5796-500ML)

2. Fetal bovine serum (FBS) (Gibco, catalog number: A5256801)

3. Penicillin-Streptomycin (Sigma-Aldrich, catalog number: P4333)

4. Phosphate-buffered saline (PBS) tablets (Sigma-Aldrich, catalog number: P4417)

5. Trypsin 10× (Sigma-Aldrich, catalog number: T4174-100ML)

6. Nocodazole (Sigma-Aldrich, catalog number: SML1665-1ML)

7. Sodium phosphate monobasic (Sigma-Aldrich, catalog number: 342483)

8. Sodium chloride (Fisher Scientific, catalog number: 10316943)

9. Sodium dodecyl sulfate (SDS) (Sigma-Aldrich, catalog number: 436143)

10. Sodium deoxycholate (Sigma-Aldrich, catalog number: D6750)

11. Ethylenediaminetetraacetic Acid (EDTA) (Fisher Scientific, catalog number: BP118-500)

12. Triton^®^ X-100 (Promega, catalog number: H5141)

13. N-Ethylmaleimide (NEM) (Sigma-Aldrich, catalog number: 04260)

14. PhosSTOP^TM^ (Roche, catalog number: 4906837001)

15. cOmplete^TM^, Mini, EDTA-free protease inhibitor cocktail (Roche, catalog number: 11836170001)

16. 1,4-dithio^TM^ threitol (DTT) (Roche, catalog number: 10197777001)

17. Tris-HCL (Trizma^®^ hydrochloride) (Sigma-Aldrich, catalog number: T5941)

18. Urea (Promega, catalog number: V3171)

19. Glycerol (Fisher, catalog number: G/0650/08)

20. Bromophenol blue (Acros, catalog number: 403140050)

21. 2-Mercaptoethanol (Sigma-Aldrich, catalog number: M6250)

22. Pierce^TM^ protein A/G agarose (Thermo Scientific, catalog number: 20421)

23. SUMO1 antibody (Abcam, catalog number: ab32058)

24. SUMO2/3 antibody (Abcam, catalog number: ab81371)

25. Hydrochloric acid (HCl), 32% (required to adjust pH) (Fisher Scientific, catalog number: 10458980)

26. Sodium hydroxide (NaOH) (required to adjust pH) (Merck, catalog number: 1064621000)

27. Tris-Glycine-SDS PAGE buffer 10× (National diagnostics, catalog number: EC-869)

28. Novex Tris-Glycine Mini Protein Gels (Life Technologies Ltd., catalog number: XP04205BOX)

29. PageRuler^TM^ Plus Prestained Protein Ladder (ThermoFisher Scientific, catalog number: 26619)

30. Tris-Glycine electroblotting buffer 10× (National diagnostics, catalog number: EC-880)

31. PVDF membrane 0.45 μm pore (VWR, catalog number: 10600023)

32. Methanol (VWR, catalog number: 20847.307)

33. Skimmed milk powder (Millipore, catalog number: 70166)

34. Tween 20 (VWR, catalog number: 663684B)

35. Polyclonal swine anti-rabbit immunoglobulins HRP (Agilent Dako, catalog number: P0217)

36. Polyclonal rabbit anti-mouse immunoglobulins HRP (Agilent Dako, catalog number: P0260)

37. ECL western blotting substrate (Promega, catalog number: W1015)

38. RANGAP1 polyclonal antibody (Invitrogen, catalog number: PA5-92435)

39. (Optional) Pierce^TM^ 660 nm protein assay (ThermoFisher Scientific, catalog number: 22660)

40. (Optional) ionic detergent compatibility reagent (ThermoFisher Scientific, catalog number: 22663)

41. (Optional) Cyclin B1 antibody (Cell Signaling, catalog number: 12231)

42. (Optional) α Tubulin antibody (Novus Biologicals, catalog number: NB100-690)

43. (Optional) Histone H3 (Cell Signaling, catalog number: 9715)

**Solutions**

1. Cell growth medium (see Recipes)

2. 1× PBS (see Recipes)

3. 1× Trypsin (see Recipes)

4. 10 M NaOH (see Recipes)

5. 1 M sodium phosphate, pH 7.4 (see Recipes)

6. 1% SDS lysis buffer (see Recipes)

7. 0% SDS lysis buffer (see Recipes)

8. 0.1% SDS buffer (see Recipes)

9. 1 M DTT (see Recipes)

10. 1 M Tris, pH 6.8 (see Recipes)

11. Loading buffer (see Recipes)

12. 1× running buffer (see Recipes)

13. 1× transfer buffer (see Recipes)

14. PBST 0.1% (see Recipes)

15. 5% milk (see Recipes)

**Recipes**

**1. Cell growth medium**

**Table:** 

Reagent	Final concentration	Quantity or volume
Dulbecco’s modified Eagle’s medium, high glucose		445 mL
FBS	10%	50 mL
Penicillin-streptomycin	1%	5 mL

**2. 1× PBS**

**Table:** 

Reagent	Final concentration	Quantity or volume
PBS	0.01 M phosphate buffer, 0.0027 M potassium chloride, and 0.137 M sodium chloride, pH 7.4, at 25 °C	2 tablets
ddH_2_O		400 mL

Autoclave 1× PBS solution to sterilize.

**3. 1× Trypsin**

**Table:** 

Reagent	Final concentration	Quantity or volume
10× trypsin	1×	5 mL
1× PBS		45 mL

**4. 10 M NaOH**

**Table:** 

Reagent	Final concentration	Quantity or volume
NaOH	10 M	40 g
ddH_2_O		100 mL

**5. 1 M sodium phosphate, pH 7.4**

**Table:** 

Reagent	Final concentration	Quantity or volume
Sodium phosphate monobasic	1 M	69 g
ddH_2_O		Up to 500 mL

Adjust pH to 7.4.

**6. 1% SDS lysis buffer pH 7.4**

**Table:** 

Reagent	Final concentration	Quantity or volume
1 M sodium phosphate, pH 7.4	20 mM	10 mL
Sodium chloride	150 mM	4.38 g
SDS	1% w/v	5 g
Sodium deoxycholate	0.5% w/v	2.5 g
EDTA	5 mM	730.6 mg
Triton X-100	1%	5 mL
ddH_2_O		Up to 500 mL
Directly before use, dissolve the following into 10 mL of the stock solution listed above		
NEM	10 mM	12.5 mg
cOmplete^TM^, Mini, EDTA-free protease inhibitor cocktail	1×	1 tablet
PhosSTOP^TM^	1×	1 tablet

In aqueous solutions, NEM and inhibitor cocktails are unstable. Excess solution containing inhibitors can be aliquoted and stored at -20 °C for up to a month.

**7. 0% SDS lysis buffer pH 7.4**

**Table:** 

Reagent	Final concentration	Quantity or volume
1 M sodium phosphate pH 7.4	20 mM	10 mL
Sodium chloride	150 mM	4.38 g
Sodium deoxycholate	0.5%	2.5 g
EDTA	5 mM	730.6 mg
Triton X-100	1%	5 mL
ddH_2_O		Up to 500 mL
Directly before use, dissolve the following into 10 mL of the stock solution listed above		
NEM	10 mM	12.5 mg
cOmplete^TM^, Mini, EDTA-free protease inhibitor cocktail	1×	1 tablet
PhosSTOP^TM^	1×	1 tablet

In aqueous solutions, NEM and inhibitor cocktails are unstable. Excess solution containing inhibitors can be aliquoted and stored at -20 °C for up to a month.

**8. 0.1% SDS buffer pH 7.4**

**Table:** 

Reagent	Final concentration	Quantity or volume
1% SDS lysis buffer		2 mL
0% SDS lysis buffer		18 mL

**9. 1 M DTT**

**Table:** 

Reagent	Final concentration	Quantity or volume
DTT	1 M	1.54 g
ddH_2_O		Up to 10 mL

Store in dark conditions at -20 °C.

**10. 1 M Tris, pH 6.8**

**Table:** 

Reagent	Final concentration	Quantity or volume
Tris-HCl	1 M	157.60 g
ddH_2_O		Up to 1 L

Adjust pH solution to 6.8.

**11. Loading buffer**

**Table:** 

Reagent	Final concentration	Quantity or volume
SDS	8% w/v	8 g
Urea	6 M	36 g
Glycerol	40%	40 mL
1 M Tris-HCl, pH 6.8	0.2 M	20 mL
2-Mercaptoethanol	5%	5 mL
Bromophenol blue		400 mg

Open and dispense 2-mercaptoethanol in a chemical safety hood. To maximize the reducing capability of 2-mercaptoethanol, aliquots of loading buffer should be stored at -20 °C. Alternatively, 2-mercaptoethanol can be supplemented at 5% concentration into a volume of loading buffer directly before use.

**12. 1× running buffer**

**Table:** 

Reagent	Final concentration	Quantity or volume
Tris-Glycine-SDS PAGE buffer 10×	1×	100 mL
ddH_2_O		Up to 1 L

**13. 1× transfer buffer**

**Table:** 

Reagent	Final concentration	Quantity or volume
Tris-glycine electroblotting buffer 10×	1×	100 mL
Methanol	20%	200 mL
ddH_2_O		Up to 1 L

**14. PBST 0.1%**

**Table:** 

Reagent	Final concentration	Quantity or volume
PBS	1×	5 tablets
Tween 20	0.1%	1 mL
ddH_2_O		1 L

**15. 5% milk**

**Table:** 

Reagent	Final concentration	Quantity or volume
Dried skimmed milk powder	5% w/v	2.5 g
PBST 0.1%		Up to 50 mL

**Laboratory supplies**

1. 150 mm^2^ cell culture dish, tissue culture treated (Corning, catalog number: 430599)

2. 25 and 10 mL pipettes (Corning Costar, catalog numbers: CLS4250, CLS4488)

3. 50 mL centrifuge tubes (Corning, catalog number: CLS430921-500EA)

4. Cell counting chambers (Biosigma, catalog number: 390497)

5. 1.5 mL microtubes (Axygen, catalog number: MCT-150-C)

6. 10, 200, and 1,000 μL pipette tips (Starlab, catalog numbers: S1111-3700, S1111-1716, S1111-6811)

7. Whatman 3 MM chromatography paper (Cytiva, catalog number: 3030-917)

8. Cling film (Fisher Scientific, catalog number: 12872233)

9. X-ray film 18 × 24 cm (Scientific Laboratory Supplies, catalog number: MOL7016)

## Equipment

1. CO_2_ incubator (Thermo Scientific, model: BB15)

2. Biosafety cabinet (Biopharma group, model: SafeFAST Classic 212 D)

3. Pipette filler (Thermo Scientific, catalog number: 9531)

4. Water bath (Grant Instruments, model: SAP18)

5. Light microscope (Olympus, model: CK30)

6. Large benchtop centrifuge (Thermo Scientific, model: Heraeus Megafuge 40)

7. Refrigerated benchtop centrifuge (Eppendorf, model: 5430 R)

8. Heat block (Labnet International, model: D1100)

9. Sonicator (Misonix, model: XL2000)

10. Vortex (Labnet International, model: S0200-230V-UK)

11. Roller (Stuart, model: SRT9D)

12. Minigel tank (Invitrogen, catalog number: A25977)

13. Powerpack (Fisherbrand, model: Powerpro-300)

14. Transfer tank (Hoefer, model: TE62)

15. Automatic X-ray film processor (Protec, model: Optimax)

16. 18 × 24 cm X-ray film cassette (Cytiva, model: RPN 11642)

17. pH meter (HANNA instruments, model: HI-2020-02)

18. Manual pipettes (0.5–10 µL, 20–200 µL, 100–1,000 µL) (Starlab, catalog numbers: S7100-0510, S7100-2200, S7110-1000)

19. (Optional) Multiskan SkyHigh microplate spectrophotometer (Thermo Scientific, model: A51119600C)

## Software and datasets

1. ImageJ (National Institute of Health, 1.54i) [29]

## Procedure

**A. Cell culture and mitotic shake-off**

1. Plate 2 million U2OS cells into each 4 × 150 mm^2^ cell culture dish and culture in a humidified atmosphere at 37 °C with 5% CO_2_ for 3 days, until a cell confluency of 70%–80% is achieved.

*Note: The number of dishes required will depend on the number of conditions tested. Here, we describe the basic experimental setup to assess basal mitotic SUMO conjugation in a cell line. This can be easily scaled and adapted to address specific research questions. The precise number of cells and dishes needed will depend on the cell line under investigation.*

2. Once cells have reached 70%–80% confluency, in the evening, remove the media from the cells and add 25 mL of fresh media to each dish, prewarmed to 37 °C, supplemented with 100 ng/mL nocodazole. Incubate on cells for 16 h.

3. The following morning (after 16 h), check the efficacy of mitotic enrichment using a light microscope. Mitotic cells will appear rounded. Handle the dishes carefully, as heavy bumps and knocks can cause mitotic cells to detach.

4. Collect the mitotic cells one plate at a time. To do this, draw the media from one dish up into a 25 mL pipette and hit the bottom of the dish onto a flat surface three times to begin loosening the mitotic cells. Place half of the media in the pipette into a 50 mL centrifuge tube and half back onto the cells. Using a 10 mL pipette, draw up the media on the plate and begin to gently and repeatedly wash the bottom of the dish, making sure to spray the whole surface area. We find 4–6 of these washes to be sufficient to detach all mitotic cells into suspension.

*Note: U2OS cells can be particularly hard to shake off, purely through the means of tapping the dish. We found that gentle repetitive washes aid significantly in detaching mitotic cells without disturbing asynchronous cells. However, not all cell types will require this extra force. The need for washing over tapping alone and the force of the washes should be determined empirically for each cell line.*

5. Using a light microscope, check whether all rounded cells have detached from the bottom of the dish and that no peeling of the asynchronous cell population has occurred. If a significant proportion of rounded cells are still attached, continue with the washes; otherwise, collect the media with the suspended mitotic cells into the same 50 mL centrifuge tube used in step A4.

6. Harvest the mitotic cells from the second plate into the same 50 mL centrifuge tube as the one used for the first plate, so that it contains the mitotic cells from 2 × 150 mm^2^ dishes. Repeat for the remaining dishes, pooling samples under the same conditions.

*Note: For the basic setup described here, using one cell line and no additional treatments, all the dishes contain cells under similar conditions and can be pooled. Understandably, when adapting/expanding this protocol to include different treatments and/or cell lines, only similar conditions should be pooled.*

7. Once all samples have been collected, mix the samples by inverting the tubes. Take 10 μL of each cell suspension and load into a cell counting chamber. Obtain the cell count for each sample and adjust the volume of each cell suspension to ensure an even number of cells across all samples.

*Note: From this point forward, all samples should be treated identically to retain an equal quantity of starting material established here.*

8. Pellet the cells using a benchtop centrifuge set to 300× *g* for 5 min. Remove the media from the pellet and tap the tube on the benchtop to loosen it. Resuspend each cell pellet in 1 mL of PBS.

9. Transfer the cell suspensions to labeled 1.5 mL microtubes and centrifuge samples at 300× *g* for 5 min in a refrigerated benchtop centrifuge, set to 4 °C. Remove PBS from the resulting cell pellet and proceed to the following steps.

**Pause point:** Cell pellets can be snap frozen and stored short-term (<2 weeks) in a -80 °C freezer at this point. Thaw samples on ice to proceed with the protocol.

**B. Preparation of cell lysate**

1. Disrupt the cell pellets by flicking the tube with your finger and resuspend in 200 μL of 1% SDS lysis buffer. Place on ice for 10 min.

*Note: Upon the addition of the lysis buffer, the sample will become very viscous. If care is not taken, the lysate can become stuck in the pipette.*

**Pause point:** Lysates can be snap frozen and stored short term (<2 weeks) in a -80 °C freezer at this point. Thaw samples on ice to proceed with the protocol.

2. Sonicate the lysate by subjecting the sample to 5 s of continuous sonication and place on ice for 10 s. Repeat this process three more times so that the sample has received 4 × 5 s of sonication. The viscous sample should be fluid by the end of this process.

*Note: We use a Microson XL 2000 ultrasonic liquid processor, tuned to vibrate at a fixed frequency of 22.5 kHz, and use a probe intensity setting of 10 to perform this step. However, other devices should perform equally as well.*

3. To denature the proteins in the lysate, add 50 mM DTT to each sample (10 μL of a 1 M DTT stock solution) and incubate on ice for 30 min.

4. Centrifuge the lysate at 14,000× *g* for 15 min in a refrigerated benchtop centrifuge, set to 4 °C, to clarify the sample.

5. Remove 30 μL of the supernatant from each tube and place into a single labeled microcentrifuge tube containing 60 μL of loading buffer. Briefly mix using the pipette tip before boiling for 5 min at 95 °C. Store at -20 °C until the following day. This will be run as the input for the immunoprecipitation.

*Note: Due to the reduced level of SUMOylation in mitosis compared to asynchronous cells, taking an input before diluting the sample will ensure that a sufficient signal for visualization is achieved from input lanes.*

6. Quantify the volume of the remaining supernatant, as volume can be lost during sonication and through handling. Ensure all samples contain equal volumes of lysate.

*Note: We found that, when performing this assay on multiple samples, 150 μL is consistently retained between all conditions. Therefore, this volume of lysate will be assumed from this point forward. However, if the minimum volume of lysate in your samples is different, we recommend retaining as much lysate as possible and recalculating the volumes for your experiment. Ensuring an equal volume between all samples is required to keep the relative amount of starting material consistent and is vitally important when comparing samples from different conditions.*

7. Dilute each sample 1:10 with ice-cold 0% SDS lysis buffer. This will result in 1.5 mL of lysate with a final SDS concentration of 0.1%.

8. Briefly mix the lysate using a 1 mL pipette tip and centrifuge at 14,000× *g* for 15 min in a refrigerated benchtop centrifuge set to 4 °C.

*Note: The equalization of cell numbers in step A7 and maintenance of consistent volumes between lysates should mean that there is an equal quantity of starting material in every sample. However, at this point, protein concentrations in the lysate can be determined for additional verification of protein content. This may be useful for downstream applications that are more sensitive than western blots, such as mass spectrometry. We have previously used the Pierce 660 nm protein assay with ionic detergent compatibility reagent, following the manufacturers’ guidelines to perform this using the Multiskan SkyHigh microplate spectrophotometer. Protein concentrations that lie within the range of 500–1,500 μg/mL have successfully been tested in this assay, producing visible SUMO smears on a western blot, although concentrations sitting at the lower end of this range will result in reduced efficiency and need longer exposure times to visualize.*

9. Combine the supernatant from both tubes into a single 15 mL centrifuge tube and store samples on ice until the agarose beads are prepared.

*Note: To keep the volumes of lysate compatible with 1.5 mL tubes and commonly available benchtop centrifuges, lysates are prepared in separate tubes and pooled into larger centrifuge tubes at the end. This is not obligatory if more convenient options are available to you.*

**C. Preparation of agarose beads and antibody conjugation**

*Note: For efficiency, the preparation of agarose beads described in this section can be run concurrently with the cell lysis described in section B.*

1. Briefly vortex Pierce protein A/G agarose beads for 2–3 s, on a middle speed setting, to achieve an even suspension.

2. Load a 200 μL pipette tip onto a pipette and, using scissors, cut the end of the tip to increase its aperture. With this tip, aliquot 30 μL of beads into 3 × 1.5 mL microfuge tubes.

3. Pipette 300 μL of 0.1% SDS buffer onto the beads and vortex, as before, to mix. Centrifuge at 500× *g* for 1 min in a benchtop centrifuge and remove the liquid from the bead pellet, leaving approximately 10–20 μL of buffer behind. Be careful not to disturb the pellet at the bottom of the tube. Repeat this wash step two more times.

4. Remove the buffer from the beads and replace with a further 1 mL of 0.1% SDS buffer. Briefly vortex the samples, as previously, to resuspend the beads.

5. To one tube of bead suspension, add 3 μg of SUMO1 antibody. To a second tube of bead suspension, add 3 μg of SUMO2/3 antibody. Leave the remaining tube of beads without antibody so it can act as a negative, bead-only control for the immunoprecipitation.

*Note: Other SUMO1 and SUMO2/3 antibodies are available. Our choice of antibodies, described here, is based on [30].*

6. Place the microtubes into a 50 mL centrifuge tube and agitate on a roller set to 15–25 rpm for 1 h, at room temperature, to allow the antibodies to bind to the beads.

7. Centrifuge the microtubes containing the beads for 1 min at 500× *g*. Remove the supernatant from the bead pellet and proceed directly to the next step.

**D. Immunoprecipitate SUMO conjugates from prepared lysates**

1. Split the prepared lysate from section B between the beads prepared in section C by adding 1 mL of lysate to each tube.

2. Place the tubes in a 50 mL centrifuge tube and incubate at 4 °C, on a roller set to 15–25 rpm, overnight.

3. The next day, pellet the beads from the lysate by centrifuging samples at 500× *g* for 1 min in a refrigerated benchtop centrifuge set to 4 °C. Discard the supernatant, being careful not to disturb the bead pellet.

4. Wash the excess, unbound proteins from the beads by adding 500 μL of ice-cold 0.1% SDS buffer to the bead pellet. Invert the sample 2–3 times to resuspend the pellet before agitating the samples on a roller set to 15–25 rpm, for 3 min, at room temperature. Centrifuge the sample at 500× *g* for 1 min and remove the buffer from the bead pellet. Repeat this wash step two more times.

5. Carefully remove the buffer from the beads, leaving as little residual liquid as possible. Add 30 μL of loading buffer directly to the bead pellet and mix using a vortex or flicking the tube with your finger.

6. Boil the samples in a heat block set to 95 °C for 10 min.

*Note: A weighted heat-resistant item can be placed on top of the tubes whilst boiling to prevent the lids popping open due to pressure.*

7. Whilst samples are boiling, remove the input sample, created in section B, from the freezer and allow it to thaw.

8. Centrifuge the boiled beads and input sample at 14,000× *g* for 5 min at room temperature.

**E. Electrophoresis and western blotting**

1. Assemble 2× Novex 4%–20% Tris-Glycine mini protein gels into a mini gel tank and cover with 1× running buffer.

*Note: The use of a gradient gel allows for the retention of free SUMO bands on the gel whilst still providing a good separation in the high-molecular-weight SUMO smear.*

**Figure 1.**
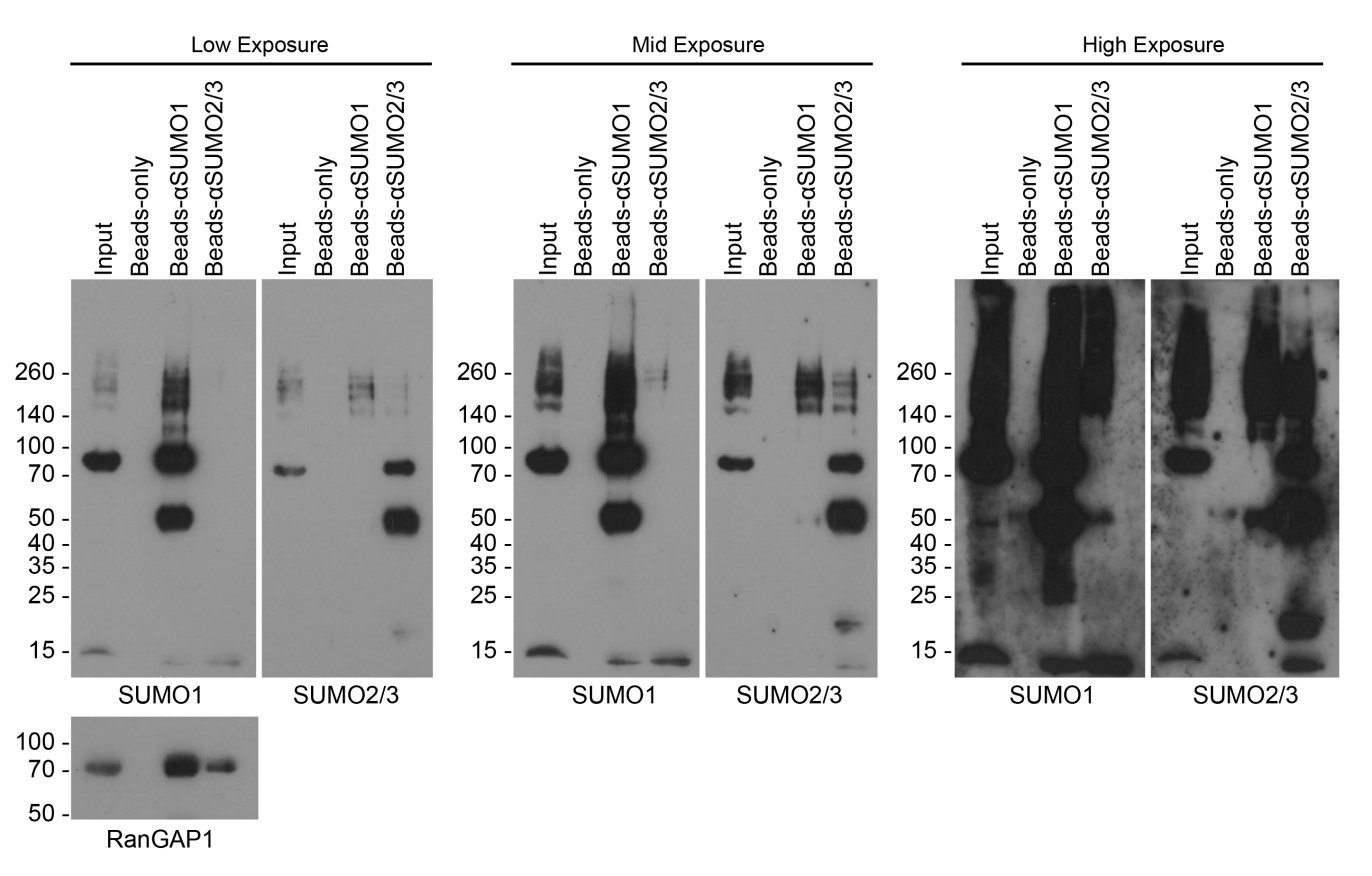
Western blots for SUMOylated proteins after denaturing SUMO immunoprecipitation. Blots show inputs for immunoprecipitation, bead-only control, and A/G beads bound with αSUMO1 (ab32058) or αSUMO2/3 (ab81371). Blots probed for SUMO1 and SUMO2/3 are presented as low (left), medium (middle), and high (right) exposures. SUMO1 conjugates appear in αSUMO2/3-IP and vice versa due to SUMO1-SUMO2/3 mixed chain conjugation [22]; antibody cross-reactivity is also a potential cause of this and should not be ruled out without empirical validation [30]. The prominent 50 kDa bands in the SUMO-IP lanes correspond to the IgG heavy chain. Western blots were also probed for the model SUMO1 substrate, RanGAP1 [1]. Shown are representative western blots from 2 experimental replicates.

2. Load the first lanes of all gels with 5 μL of protein ladder and 15 μL of input. To one gel, load half the supernatant from the negative bead-only control, SUMO1, and SUMO2/3 bead conditions (see Figure 1), being careful to avoid the bead pellet at the bottom of the tube. Repeat this for the second gel.

*Note: An excess of input sample is generated in the protocol so that a further gel can be run and probed for additional controls as desired. This becomes useful when comparing multiple samples with or without treatments.*

3. Run the gels at a constant 220 V for 45 min.

4. Activate a 7 × 9 cm rectangle of PVDF membrane with methanol and set up a transfer cassette.

5. In a cold room or a bucket of ice, transfer the gel for 3 h at 100 V using prechilled 1× transfer buffer.

*Note: We also use ice packs in the transfer tank. However, if this is not possible, placing the tank in a large bucket of ice with precooled buffer should be sufficient.*

6. Place the membranes for the SUMO1 and SUMO2/3 immunoprecipitations into two separate 50 mL centrifuge tubes containing 5 mL of 5% milk in PBST. Incubate for 30 min on a roller set to 15–25 rpm at room temperature.

7. Add 5 μL of SUMO1 antibody to one tube and 5 μL of SUMO2/3 antibody to the other. Incubate the primary antibodies on membranes at 4 °C, on a roller set to 15–25 rpm, overnight.

*Note: We have described the process of probing membranes for SUMO1 or SUMO2/3 to visualize global SUMO conjugates. This is of particular value when first performing this assay to assess immunoprecipitation efficiency, antibody sensitivity, and specificity. However, these membranes can be probed with different antibodies to try and identify possible SUMOylated substrates. See General note 6.*

8. Remove the primary antibody and wash the membranes on a roller, set to 15–25 rpm, for 5 min in 5 mL of PBST. Repeat this wash three times.

9. Dilute the appropriate HRP-conjugated secondary antibodies 1:5,000 in 5% milk and incubate on the washed membranes for 1 h at room temperature, whilst rolling at 15–25 rpm.

10. Remove the secondary antibodies and wash the membranes on a roller, set to 15–25 rpm, for 5 min in 5 mL of PBST. Repeat this wash three times.

11. On the final wash of the membrane, mix the ECL solutions as per the manufacturer’s guidelines; 1 mL of ECL is sufficient to cover one full membrane measuring 7 × 9 cm.

12. Place the washed membranes face up on a clean or cling-filmed flat surface and cover evenly with the ECL mix.

13. Dab off any excess ECL and place the membranes face down on a smooth, flat, crease-free section of cling film. Seal the back of the membranes with cling film, ensuring there are no bubbles or crinkles.

14. Stick the cling-filmed membranes into a developing cassette.

15. In a dark room, expose X-ray films to the membranes and develop using an X-ray film processor.

*Note: The duration of exposure needed to obtain a signal will be specific to the antibodies and ECL used. This will need to be worked out empirically. We perform an instant (on/off, i.e., less than 20 s), short (1–2 min), medium (5–10 min), and long (20–30 min) exposure for development. On rare occasions, an overnight exposure can be used, but this should not be required for the assay as described here.*

16. Label the X-ray films and scan into a computer as a TIFF.

17. Image J can be used to quantify SUMO smears [29] or bands, if desired.

## Data analysis

The number of biological repeats required for this assay and data analysis depends on downstream applications. Visual assessment of western blots is described here; however, quantification on western blots can be performed. One biological repeat can be used for visual assessment of pull-down efficiency and antibody specificity, and to provide an indicative result, in situations where further experimentation will be used to support findings. Robust quantification of western blot data produced by this assay requires three biological repeats.

## Validation of protocol

This protocol is an optimized version of the one used and validated, with the adaptations listed below, in the following research article:

• Lanz et al. [7]. HDAC6-dependent deacetylation of SAE2 enhances SUMO1 conjugation for mitotic integrity. *EMBO J*. (Figure 3A and EV4F).

The adaptations used for this research article were applied to address the specific research question under investigation (not fundamental to the assay’s performance). These adaptations were the use of doxycycline-inducible cell lines that expressed SAE1:SAE2 constructs resistant to the SUMO E1 inhibitor ML792, the concurrent addition of doxycycline and ML792 with nocodazole treatment, and the release of nocodazole into fresh media containing doxycycline and ML792 for 10 min before harvesting. In Figure 3A from [7], the immunoprecipitation for SUMO1 was performed using the antibody described here, but the western blot was probed using ab133352. Free SUMO1 in Figure EV4F from [7] was detected by using ab320580, as it can detect both free and conjugated forms of SUMO1.

**Figure 2.**
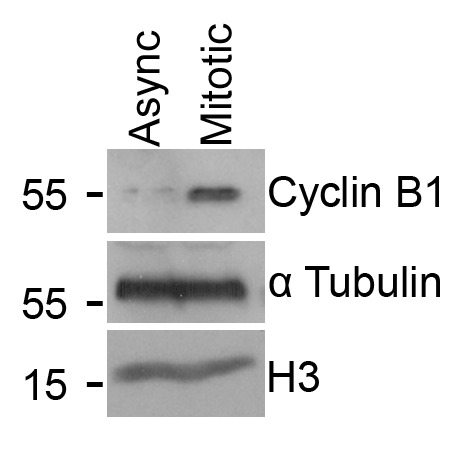
Validation of mitotic enrichment following nocodazole treatment and shake-off. The blot displays Cyclin B1 levels in lysates made from either an asynchronous cell population (Async) or cells treated with 100 ng/mL nocodazole for 16 h and harvested via mitotic shake-off (Mitotic). α Tubulin and Histone H3 are included as loading controls. This representative image was obtained from one biological repeat.

Validation of mitotic cell enrichment, following mitotic shake-off (described in section A), was performed via western blotting (Figure 2).

## General notes and troubleshooting

**General notes**

1. The concentration of nocodazole and the duration of its incubation on cells will vary depending on the cell line. For U2OS cells, a concentration of 100 ng/mL for 16–18 h achieves successful enrichment of the mitotic population with minimal cell death and/or detachment from the bottom of the plate. For U2OS cells, a good synchronization will result in approximately 30% of the cells adopting a rounded morphology. If there is a high proportion of floating cells, the concentration of nocodazole may need to be lowered.

2. This protocol can also be performed on cells released into mitosis. To release cells for a particular time into mitosis, remove all the media from the dishes and carefully rinse the plate with PBS, prewarmed to 37 °C. It is important not to add the PBS directly onto the cells but to the side of the dish to avoid mitotic cells detaching. Repeat this process two times. Once rinsed, fresh prewarmed media can be added to the plates for up to 30 min. Beyond 30 min, cells will begin to move into the later stages of mitosis, where other methods for collection are more suitable.

3. We have described the process of identifying global SUMO conjugates in a single cell line. However, this methodology can be adapted to compare SUMOylation between cell lines or after a treatment. To assess the effect of a chemical inhibitor or drug on mitotic SUMOylation, we commonly incubate the treatment on the cells in the last hour of nocodazole synchronization before harvesting the samples.

4. Many commercial SUMO1 and SUMO2/3 antibodies are available. However, they vary in their specificity and sensitivity. The antibodies chosen here have been reported to have good specificity and sensitivity in multiple applications [30]. Both antibodies can detect free and conjugated forms of SUMO. The choice of SUMO antibody for this assay is a major consideration. Different antibodies would require validation for their specificity toward different SUMO paralogs, ability to bind free and conjugated SUMO species, efficiency to immunoprecipitated substrates, and potential optimization for concentrations required.

5. We found that, for general purposes, with the protein A/G beads listed, we do not need to preclear our lysates with blank beads before immunoprecipitation. However, this could be performed if a high background is observed in negative bead-only control samples or if precipitations are to be sent for more sensitive methods of protein detection, such as mass spectrometry.

6. This protocol describes a method for precipitating SUMO conjugates from cells. However, it is also possible to perform the reciprocal of this experiment. In this scenario, antibodies to a specific target substrate are bound to beads, and the target protein is precipitated. Western blots can then be probed for SUMO1 and/or SUMO2/3 to identify the SUMOylation status of a protein of interest. This may be a more efficient means of precipitating and visualizing the modification on a low-abundance substrate.

7. For sensitive downstream applications such as mass spectrometry, peptide elution could be considered to reduce contaminants in the sample. However, for this, the antibody epitopes would need to be known or empirically determined.

8. This protocol requires a large number of cells; a mitotic shake-off on 4 × 150 mm^2^ dishes (as described in section A) will yield, in total, approximately 8 million harvested mitotic cells for lysis. This may limit the applicability of this assay to primary cells or rare samples. In such cases, it is possible to test this protocol on samples harvested from a 70%–80% confluent 10 cm^2^ dish, at a minimum, with limited success expected upon further reduction in plate area. With the reduction of the cell material, it is expected that exposure times at the western blot development stage will need to be longer, and the robustness of the data obtained is likely to be reduced.

**Troubleshooting**

**Problem 1:** Low mitotic cell numbers following nocodazole block.

Possible cause: Cell density too high or too low.

Solution: Best yields are achieved in the ~80% confluency window. Below this window, raw cell numbers become low; above the ~80% confluency window, cells may display contact inhibition, reducing cell cycling. Furthermore, at higher cell densities, cells are harder to dissociate from the plate by mitotic shake-off.

**Problem 2:** Asynchronous cells are detaching when performing a mitotic shake-off.

Possible causes: Cell density is too high, there is too much force in the washes used to shake off the mitotic cells, or the cell type requires a more gentle shake-off approach.

Solutions: Make sure cells are within the ~80% confluency window. Use a lower setting or less finger pressure on the pipette gun to lower the force of the washes. If a different cell type with weak adhesion is being used, mitotic shake-off can be performed by hitting the side of the dish, without repeated media washing.

**Problem 3:** Inconsistent mitotic enrichment between plates and/or repeats.

Possible causes: Variation in confluency between plates/repeats. Nocodazole was not homogeneously distributed across the plate when it was added.

Solutions: Ensure consistent cell confluency across plates/repeats. In our experience, the nocodazole block works best if the 100 ng/mL nocodazole media is prepared in a fresh, clean Falcon and used to replace the media on the cells. This ensures homogeneity of the 100 ng/mL nocodazole in media, which may not be the case if nocodazole is pipetted directly onto the existing media in the dish.

**Problem 4:** During western blot development, the ECL-treated HRP film exposure time is longer than expected.

Possible causes: Low protein abundance; SUMO conjugates are reduced in mitosis compared to interphase [5].

Solutions: Increase raw cell numbers by plating in larger or more numerous dishes. It may also be an option to transfect a construct expressing your protein-of-interest or a construct expressing SUMO1/2/3 to increase the SUMO conjugation. Alternatively, a highly sensitive chemiluminescent detection reagent can be used to detect lower-abundant proteins.

**Problem 5:** Protein degradation or loss of protein SUMO conjugates.

Possible cause: Limited inhibitor efficacy.

Solutions: Ensure cOmplete protease and cysteine-specific protease inhibitor (NEM) are functional and abundant. Both are light sensitive; so, as always, fresh is best. Furthermore, higher concentrations than expected may be required due to the high number of cells required to detect mitotic SUMO conjugates.

**Problem 6:** Nonspecific or “sticky” pull-down observed in beads-only control.

Possible cause: Inherent binding properties of the A/G beads.

Solutions: Use 5%–20% BSA in 0.1% SDS RIPA buffer to block A/G beads after SUMO antibody binding. Incubate beads in this buffer for ≥1 h at 4 °C with agitation. Another option is to preclear lysates with washed unconjugated A/G beads before the lysates are exposed to A/G beads conjugated with the SUMO antibody.

**Problem 7:** Streaky/poor running input samples on the SDS page.

Possible causes: The high protein concentration in the input can overload the well, causing poor band resolution. Alternatively, the sample is not sufficiently sonicated.

Solutions: If the problem is due to protein concentration, try diluting the input before loading and/or loading less into the gel. Inputs can be taken from the 1:10 diluted lysate, but due to the reduced levels of SUMOylation in mitosis, SUMO smear inputs can be hard to visualize if performed in this manner. If the problem is due to insufficient sonication, the samples will appear “heavy” when loading the gel. To solve this, repeat the sonication step. This protocol ensures that there is an excess volume of input collected so that these samples can be rerun if a problem occurs.
